# Salivary lactate and 8-isoprostaglandin F_2α_ as potential non-invasive biomarkers for monitoring heart failure: a pilot study

**DOI:** 10.1038/s41598-020-64112-2

**Published:** 2020-05-04

**Authors:** Silvia Ghimenti, Tommaso Lomonaco, Francesca G. Bellagambi, Denise Biagini, Pietro Salvo, Maria G. Trivella, Maria C. Scali, Valentina Barletta, Mario Marzilli, Fabio Di Francesco, Abdelhamid Errachid, Roger Fuoco

**Affiliations:** 1 Department of Chemistry and Industrial Chemistry, University of Pisa, Via Giuseppe Moruzzi 13, 56124 Pisa, Italy; 2 Univ Lyon, CNRS, Universitè Claude Bernard Lyon 1, Institut des Sciences Analytiques, UMR 5280, 5 rue de la Doua, F-69100 Villeurbanne, France; 3 Institute of Clinical Physiology, CNR, Via Giuseppe Moruzzi 3, 56124 Pisa, Italy; 4 Department of Surgical, Medical and Molecular Pathology and Critical Care Medicine, University of Pisa, Via Paradisa 2, 56124 Pisa, Italy

**Keywords:** Biomarkers, Analytical chemistry

## Abstract

Heart failure (HF) is a cardiovascular disease affecting about 26 million people worldwide costing about $100 billons per year. HF activates several compensatory mechanisms and neurohormonal systems, so we hypothesized that the concomitant monitoring of a panel of potential biomarkers related to such conditions might help predicting HF evolution. Saliva analysis by point-of-care devices is expected to become an innovative and powerful monitoring approach since the chemical composition of saliva mirrors that of blood. The aims of this study were (i) to develop an innovative procedure combining MEPS with UHPLC-MS/MS for the simultaneous determination of 8-isoprostaglandin F_2α_ and cortisol in saliva and (ii) to monitor lactate, uric acid, TNF-α, cortisol, α-amylase and 8-isoprostaglandin F_2α_ concentrations in stimulated saliva samples collected from 44 HF patients during their hospitalisation due to acute HF. Limit of detection of 10 pg/mL, satisfactory recovery (95–110%), and good intra- and inter-day precisions (RSD ≤ 10%) were obtained for 8-isoprostaglandin F_2α_ and cortisol. Salivary lactate and 8-isoprostaglandin F_2α_ were strongly correlated with NT-proBNP. Most patients (about 70%) showed a significant decrease (a factor of 3 at least) of both lactate and 8-isoprostaglandin F_2α_ levels at discharge, suggesting a relationship between salivary levels and improved clinical conditions during hospitalization.

## Introduction

Heart failure (HF) is a complex disorder characterized by a reduced ability of the heart to maintain an adequate cardiac output (CO), which is essential to deliver oxygen to tissues and organs^1^. According to statistics, at present about 26 million people worldwide are treated for HF symptoms and it is projected that by 2030 more than 8 million adults will be diagnosed with HF because of increasing life expectancy and growing population^2^. HF is one of the most frequent cause of hospitalization in elderly people^3^, with a high rate of readmissions within 30 days post-discharge^4^. Growing numbers and frequent hospitalizations transform this pathology in a huge economic issue for health care systems; for example, Europe and USA spend for treating HF about 2% of their annual healthcare budget^5,6^.

Nowadays, diagnosis is based on a combination of symptoms (e.g. shortness of breath and fatigue) and signs (e.g. central venous hypertension, ankle swelling, pulmonary rales) that are confirmed by biochemical markers and instrumental tests (e.g. blood tests, transthoracic Doppler 2D echocardiography, lung sonography and chest X-ray)^7^. Upon diagnosis, doctors tailor an appropriate management strategy in terms of medication, nutrition and physical activity but cannot avoid high mortality rates (e.g. 11% and 41% at 1 year and 5 years, respectively)^8,9^.

In the acute phase of HF, the body tries to maintain an adequate cardiac function by activating a large number of compensatory mechanisms including neurohormonal mechanisms^10^. Among these, hypothalamic-pituitary-adrenal axis, sympathetic and the renin-angiotensin-aldosterone systems are usually activated during HF to maintain circulatory homeostasis and blood pressure^11,12^. Cortisol and α-amylase are well-known chemicals related to these conditions and thus might be useful indicators for monitoring HF evolution^13^. Earlier studies have shown that HF is also associated with hyperlactatemia^14^, oxidative stress^15^, hyperuricemia^16^ and inflammation^17^, exacerbating the pathophysiology of the HF. Hyperlactatemia can be viewed as part of the stress response that includes an increased metabolic rate, the activation of the sympathetic nervous system, an accelerated glycolysis and a modified bioenergetic supply^14^. Blood lactate levels increase during ischemia and after ischemia-reperfusion injury due to an altered anaerobic metabolism and an inadequate tissue perfusion^18,19^. Moreover, several studies have highlighted that oxidative stress (OS), defined as an imbalance between antioxidant defenses and production of reactive oxygen species (ROS), is enhanced in HF and may cause cellular dysfunction, DNA damage, protein and lipid peroxidation^20–24^. Isoprostanes are compounds produced *in vivo* from the oxidative peroxidation of arachidonic acid and thus provides an accurate assessment of OS both *in vitro* and in vivo^25^. In the setting of HF, hyperuricemia is often associated with reduced exercise capacity, inflammation markers, endothelial dysfunction, oxidative stress and diastolic dysfunction^26^. The increased blood levels of uric acid (UA) depends of both enhanced production resulting from OS and to a decreased excretion due to renal failure^27^. Tumour necrosis factor alpha (TNF-α) is one of the cytokines involved in the pathogenesis of HF^28^, leading to cardiomyocyte, hypertrophy, fibrosis and negative inotropic effects^28,29^.

In the last decades, the unobtrusive monitoring of health conditions and drug therapies by the analysis of fluids that can be collected in a non-invasive way (e.g. breath, saliva, sweat, and wound exudate) has attracted much attention^30–34^. Saliva, whose chemical composition mirrors that of blood, can be collected in a non-invasive way by easy sampling procedures requiring some cautions^35–37^. Compared to blood and its derivatives, it is safer to handle and transport^38^, and its simpler chemical composition makes it particularly suitable for human biomonitoring in combination with POC devices^39–41^.

The aims of this study were i) to develop an innovative procedure combining micro-extraction by packed sorbent (MEPS) with ultra-high-performance liquid chromatography coupled to electrospray ionization triple-quadrupole mass spectrometry (UHPLC-ESI-MS/MS) for the simultaneous determination of 8-isoprostaglandin F_2α_ (8-isoPGF_2α_) and cortisol in saliva and ii) to monitor lactate, uric acid, TNF-α, cortisol, α-amylase and 8-isoPGF_2α_ concentrations in stimulated saliva samples collected from 44 HF patients during their hospital stay due to acute HF. We hypothesize that changes in the chemical composition of patients’ saliva during recovery of baseline conditions due to therapies are specular to changes occurring at home when patients drift towards acute conditions. Reliable biomarkers predicting HF flares are needed to develop sensing devices usable at home, from the patient themselves or caregivers, that may provide an early advice of the building up of acute conditions. This paper aims at identifying the target molecules and providing the basic knowledge needed for the development of this kind of devices.

## Results

### Development of MEPS procedure for the determination of 8-isoprostaglandin F_2α_ and cortisol in saliva

The optimization of the MEPS procedure maximized the extraction efficiency of 8-isoPGF_2α_ and cortisol in saliva samples. We investigated dilution ratio of the sample, sampling cycles, composition of the washing solution and volume of the elution solvent as possible parameters affecting MEPS performance.

Proteins, mucins and other interferences in the matrix may cause a premature deterioration of the MEPS sorbent performance and/or a cartridge occlusion^42^. To prevent these issues, saliva sample can be diluted with water and then filtered using a syringe filter prior to MEPS extraction. The influence of the sample to water dilution ratio, i.e. 1:2, 1:5 and 1:8 v/v, on the analyte peak area was investigated. For this purpose, nine aliquots (500 µL each) of pooled saliva samples, spiked with 8-isoPGF_2α_ (50 pg/mL) and cortisol (500 pg/mL), were diluted with LC-MS water to achieve the desired dilution ratio and then filtered at 0.2 µm before starting the MEPS procedure. The entire volume of each sample, namely 1500, 3000 and 4500 µL, was loaded up and discharged 3, 6 and 9 times, respectively. The target analytes were eluted with 50 µL of methanol, so that the sample aliquot volume (500 µL) to solvent elution volume ratio was fixed at 10. The filtration step and the sample to water dilution ratio did not significantly (*p* > 0.05) affect the analytes peak area, but the 1:2 v/v dilution ratio was soon excluded because of sorbent occlusion just after ten MEPS extractions. Thus, we decided to use 1:5 sample to water dilution ratio and filter the resulting aqueous solution as the best compromise in terms of duration of the MEPS procedure and cartridge lifetime.

Methanol was added to the aqueous washing solution used during the MEPS procedure^43^, and no significant analyte loss was observed when the washing step was carried out with a 95:5 v/v water:methanol mixture.

A quantitative elution of the analyte was achieved by a single withdraw-eject cycle of 50 µL of methanol, since the content of 8-isoPGF_2α_ and cortisol in the methanol used in the second withdraw-eject cycle was below the limit of detection.

### Analytical figures of merit of MEPS procedure

Five working standard solutions were analysed in triplicate to obtain calibration curves in the range 50–1250 pg/mL and 500–12500 pg/mL for 8-isoPGF_2α_ and cortisol, respectively. A known amount (20 ng/mL) of internal standard (IS), i.e. 8-iso prostaglandin F_2α_-d4 (8-isoPGF_2α_-d4), was added to these solutions before to MEPS-UHPLC-ESI-MS/MS analysis. For 8-isoPGF_2α_, calibration curves were constructed by plotting the analyte to IS peak area ratios (A/A_IS_) versus the corresponding concentration ratios (C/C_IS_), whereas for cortisol the analyte peak areas were plotted versus the corresponding concentrations. The best calibration plots (a = m × b) were evaluated using the Deming regression and the slope (average ± standard deviation) for 8-isoPGF_2α_ and cortisol resulted 1.2 ± 0.1 and (1.3 ± 0.1) × 10^4^, respectively.

The presence of matrix effect was ruled out by comparing the slopes of the calibration curves (i.e. standard solutions and spiked samples) at a confidence level of 95%.

Limit of detection (LOD) was estimated as three times the standard deviation (s.d.) of the “low level spiked blank” sample, i.e. 50 pg/mL for both 8-isoPGF_2α_ and cortisol. Each saliva sample was prepared five times and then analysed with the MEPS-UHPLC-ESI-MS/MS approach. A LOD equal to 10 pg/mL was obtained for both 8-isoPGF_2α_ and cortisol.

Standard spiked saliva samples were analysed three times within the same day and on three consecutive days to evaluate the intra- and inter-day recovery, respectively. For both analytes, the recovery was almost quantitative (95–110%) with intra- and inter-day precisions below 10%.

Six aliquots of pooled saliva samples were prepared by spiked a known amount of 8-isoPGF_2α_ (50 pg/mL) and cortisol (500 pg/mL). Three samples (S1) were analysed according to the procedure described in 4.5.2 whereas the other aliquots were absorbed in three Salivette polyester swabs to evaluate the analyte recovery from the collection device. Sample (S2) was recovered by centrifuging the device at 7000 rpm and 4 °C for 5 min and then analysed with the same method. The recovery was calculated as S2 to S1 8-isoPGF_2α_ and cortisol concentration ratio. For both analytes the mean recovery and the corresponding relative standard deviation resulted 95% and 10%, respectively.

These results confirmed that MEPS-UHPLC-ESI-MS/MS method allows a reliable quantification of 8-isoPGF_2α_ and cortisol in saliva samples.

### Stability of 8-isoprostaglandin F_2α_ solutions

Cortisol is a very stable analyte both in water solution either in saliva samples^44^. The stability of standard working solutions and saliva samples was evaluated at −80 °C, whereas the stability of extracted samples at 4 °C. The initial concentrations of the analyte (t = 0 h) were used as the reference values and stability was evaluated by analysing the variance (ANOVA) at a confidence level of 95%.

Standard working solutions of 8-isoPGF_2α_ were stable for the entire observational period (one month), whereas extracted samples in methanol were stable throughout the duration of a typical sequence of chromatographic analyses (storage in the autosampler over 24 h at 4 °C). The 8-isoPGF_2α_ concentration in saliva was stable at a confidence level of 95%, both after one week storage at 4 °C and six months at −80 °C.

### Monitoring heart failure during hospitalization: a pilot study

Thirteen (30%) out of the 44 patients enrolled had been diagnosed for HF at least 2 years before their inclusion in the pilot study, and 9 of them had one HF episode within 6 months from their first hospital admission (t_0_). Over 90% of the HF patients showed comorbidities such as diabetes mellitus (n = 17), hypertension (n = 29), dyslipidaemia (n = 22), chronic obstructive pulmonary disease (n = 9), chronic kidney disease (n = 14), chronic liver disease (n = 2), coronary artery disease (n = 18), atrial fibrillation (n = 15), and oncological diseases (n = 6). Table 1 reports the demographic and clinical data of HF patients, both at the hospital admission and discharge.

**Table 1:** Characteristics of enrolled patients (n = 44) at the hospital admission (t_0_) and discharge (t_d_).

Characteristics	Hospital admission (t_0_)	Hospital discharge (t_d_)	*p*-value
Age (years)	72 [16]		
Weight (Kg)	77 ± 20	72 ± 16	0.14
Body mass index (Kg/m^2^)	30 ± 6	26 ± 4	0.22
Heart failure severity NYHA class at admission			
NYHA I	4 (9%)		
NYHA II	8 (18%)		
NYHA III	18 (41%)		
NYHA IV	14 (32%)		
Systolic blood pressure (mmHg)	123 ± 20	119 ± 18	0.39
Diastolic blood pressure (mmHg)	75 ± 14	68 ± 13	0.07
Heart rate (bpm)	75 [26]	70 [17]	0.16
Respiratory rate (breathes per minute)	20 ± 8	16 ± 5	0.11
Left ventricular ejection fraction (%)	31 [13]	52 [12]	**0.01**
Low-density lipoprotein (mg/dL)	85 [59]	76 [52]	0.69
High-density lipoprotein (mg/dL)	42 ± 13	43 ± 12	0.79
Triglyceride (mg/dL)	93 [54]	92 [46]	0.77
Total calcium (mg/dL)	9 ± 1	9 ± 1	0.84
Sodium (mmol/L)	141 ± 7	139 ± 6	0.44
Potassium (mmol/L)	4 ± 1	4 ± 1	0.37
Haemoglobin (g/dL)	15 ± 2	13 ± 2	0.91
Haematocrit (%)	39 ± 6	38 ± 7	0.78
White blood cell count (×10^9^/L)	8 [4]	5 [2]	**0.02**
Creatinine (mg/dL)	1.5 [0.5]	0.8 [0.5]	0.38
Estimated glomerular filtration rate (mL/min)	59 ± 23	57 ± 25	0.77
Blood NT-proBNP (pg/mL)	1150 [1660]	510 [1220]	**0.01**
Alanine aminotransferase (U/L)	23 [21]	19 [12]	0.21
Aspartate aminotransferase (U/L)	20 [33]	19 [22]	0.69
Oxygen saturation (%)	92 ± 4	98 ± 3	0.18
Glycaemia (mg/dL)	110 [51]	98 [42]	0.18
Glycated haemoglobin (mmol/mol)	48 [5]	47 [6]	0.97
Salivary pH	6.9 ± 0.6	7.1 ± 0.5	0.75
Salivary flow rate (mL/min)	0.5 [0.5]	0.7 [0.3]	0.09
Salivary lactate (μM)	1670 [3160]	620 [1030]	**<0.01**
Salivary uric acid (mg/mL)	95 [110]	55 [50]	**0.02**
Salivary TNF-α (pg/mL)	25 [45]	20 [30]	0.42
Salivary cortisol (pg/mL)	760 [1560]	720 [1280]	0.07
Salivary α-amylase (U/mL)	480 [470]	400 [450]	0.78
Salivary 8-isoPGF_2α_ (pg/mL)	40 [30]	25 [21]	**0.02**

Data are shown as mean ± standard deviation or median [interquartile range, calculated as 75^th^ - 25^th^ percentiles]. Bold values denote statistical significance at the *p* < 0.05 level.

The Mann-Whitney test revealed statistically significant gender differences only for weight and body mass index (*p* < 0.05).

Compared to the hospital admission (acute phase) (Table 1), HF patients showed at discharge: i) significantly higher median (lower and upper quartiles) value of LVEF [52% (45–57%) *vs*. 31% (25–38%), *p* < 0.05], ii) lower plasmatic values of NT-proBNP [510 pg/mL (250–1470 pg/mL) *vs*. 1150 pg/mL (590–2250 pg/mL), *p* < 0.05] and white blood cell count [5 ×10^9^/L (4–6 ×10^9^) *vs*. 8 ×109/L (6–10 × 109), *p* < 0.05]. During hospitalization, a slight decrease of the mean value of systolic blood pressure [123 mmHg (87–180 mmHg) *vs*. 119 mmHg (73–150 mmHg), *p* > 0.05], the diastolic blood pressure [75 mmHg (50–150 mmHg) *vs*. 68 mmHg (38–90 mmHg), *p* > 0.05], the body weight [77 Kg (65–102 Kg) *vs*. 72 Kg (61–88 Kg), *p* > 0.05] and the BMI [30 Kg/m^2^ (26–32 Kg/m^2^) *vs*. 26 Kg/m^2^ (24–29 Kg/m^2^), *p* > 0.05] was also noticed. The same trend was also observed for the median (lower and upper quartiles) value of heart rate [75 bpm (62–88 bpm) *vs*. 70 bpm (64–81 bpm), *p* > 0.05] and glycaemia [110 mg/dL (86–137 mg/dL) *vs*. 98 mg/dL (81–123 mg/dL), *p* > 0.05]. Interestingly, a remarkable decrease (40–60%) over time during hospitalization was noticed for the median (lower and upper quartiles) value of salivary lactate [1670 µM (630–3790 µM) *vs*. 620 (190–1220 µM), *p* < 0.05], uric acid [95 mg/mL (55–160 mg/mL) vs. 55 mg/mL (90–40 mg/mL)] and 8-isoPGF_2α_ [40 pg/mL (30–60 pg/mL) *vs*. 25 (25–46 pg/mL), *p* < 0.05].

A stepwise regression analysis (Supplementary Table S1) was applied to identify the variables more closely related to the blood level of NT-proBNT, a sensitive marker of HF.

The model had the coefficient of determination (R^2^) of 0.7 and *p*-value < 0.001. The stepwise regression allowed the variables to be reduced to five. Since the literature already describes left ventricular ejection fraction, oxygen saturation and estimated glomerular filtration rate as risk or detrimental factors for HF^45–47^, we therefore focused on lactate and 8-isoPGF_2α_ as new potential predictors for monitoring HF patients.

**Figure 1:**
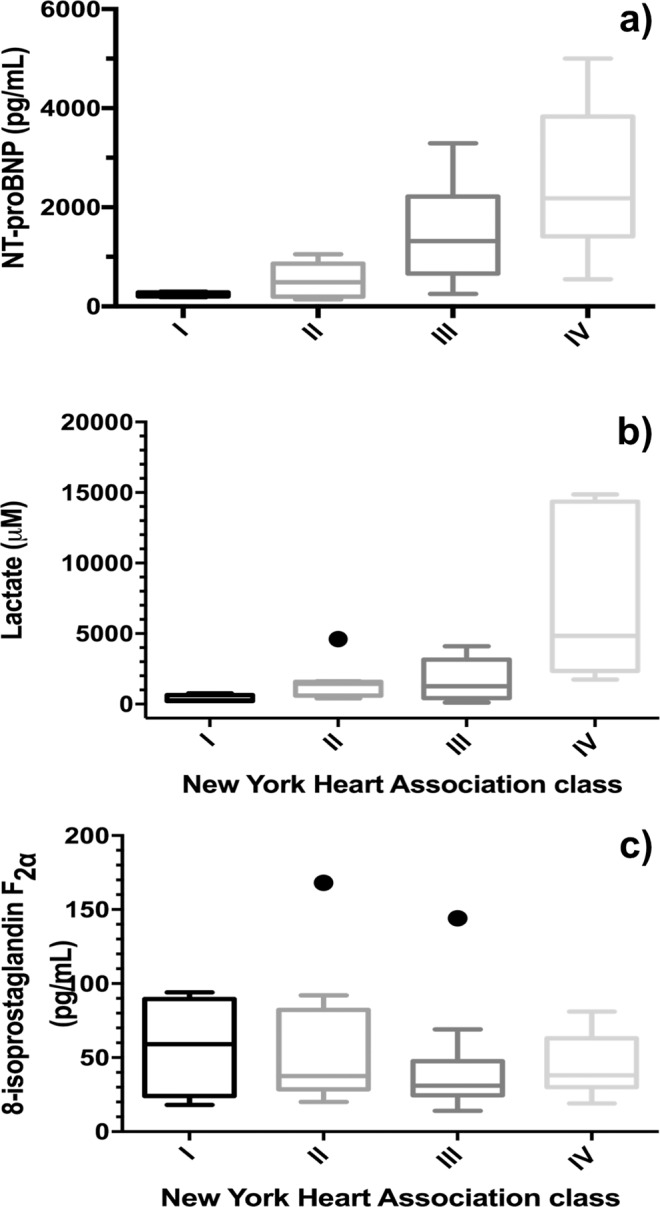
Box-plot for NT-proBNP (**a**), lactate (**b**), and 8-isoPGF_2α_ (**c**) for four NYHA functional classes. Note: The box-plot shows: the minimum, the 25^th^ percentile, the median, the 75^th^ percentiles, and the maximum value for each variable investigated. The outliers are shown with black points.

When patients were grouped in one of four categories of the NYHA functional class (Fig. 1), only NT-proBNP content in blood and salivary lactate were significantly different among the four classes (confidence level of 95%), whereas salivary 8-isoPGF_2α_ was not correlated to the NYHA classes.

Salivary levels of lactate and 8-isoPGF_2α_ levels at the admission and at the discharge were not significantly different (confidence level of 95%) when patients were grouped according to the comorbidities. As an example, median (lower and upper quartiles) salivary lactate concentrations calculated for diabetic (n = 17) and non-diabetic subgroup (n = 27) resulted 1610 μM (700–3700 μM) and 2050 μM (1250–4105 μM), respectively. In the same way, these patients showed similar salivary levels of 8-isoPGF_2α_ [35 pg/mL (25–45 pg/mL) *vs*. 40 pg/mL (25–60 pg/mL)].

**Figure 2:**
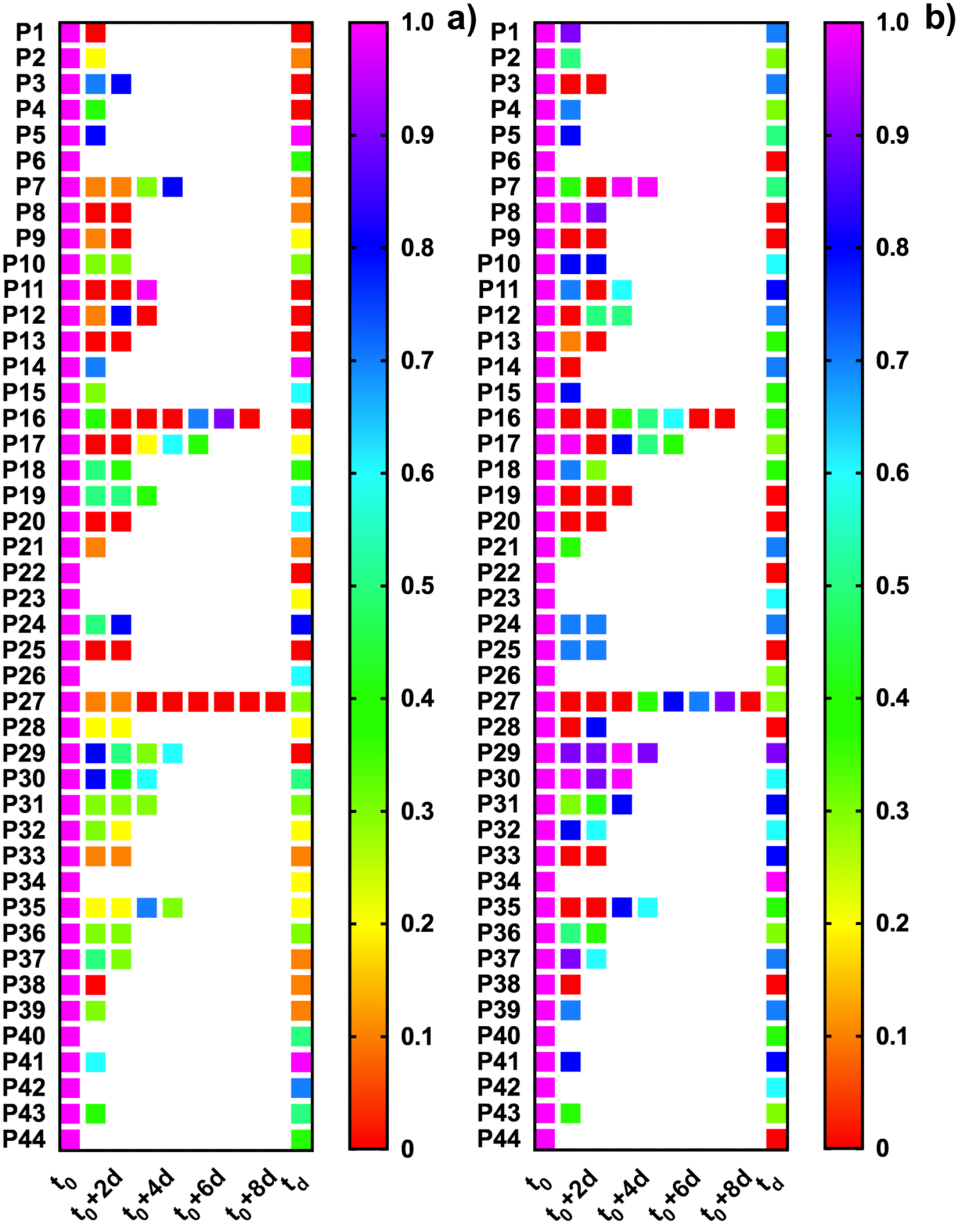
Heatmap of salivary lactate (**a**) and 8-isoPGF_2α_ (**b**) levels measured for all the enrolled patients (n = 44) during the hospitalization. Data were normalized with respect to the hospital admission point (t_0_). Each square represents a collection point.

Figure 2 shows for each patient the changes over time of salivary concentrations of lactate and 8-isoPGF_2α_, normalized with respect to the admission level.

The majority of HF patients (about 70%) had a remarkable three-fold decrease of lactate and 8-isoPGF_2α_ concentration in saliva over time (Fig. 2). Eighteen patients, belonging to NYHA class III and IV and characterized by low LVEF (20–30%) and high blood NT-proBNP (1200 pg/mL or higher) values at admission, showed a very marked decrease of lactate (5 to 10-fold). Only ten patients showed a decrease of 8-isoPGF_2α_ at least of a factor of three, and five patients had stable (within ±30%) lactate and 8-isoPGF_2α_ levels (about 500 µM and 30 pg/mL, respectively) during their stay at the hospital. Moreover, two more patients had similar results in terms of lactate levels, although they showed high 8-isoPGF_2α_ concentrations, i.e. from 50 up to 100 pg/mL. Interestingly, the decrease of both lactate and 8-isoPGF_2α_ levels was not affected by the presence of comorbidities such as diabetes mellitus, dyslipidaemia, and kidney disease.

**Figure 3:**
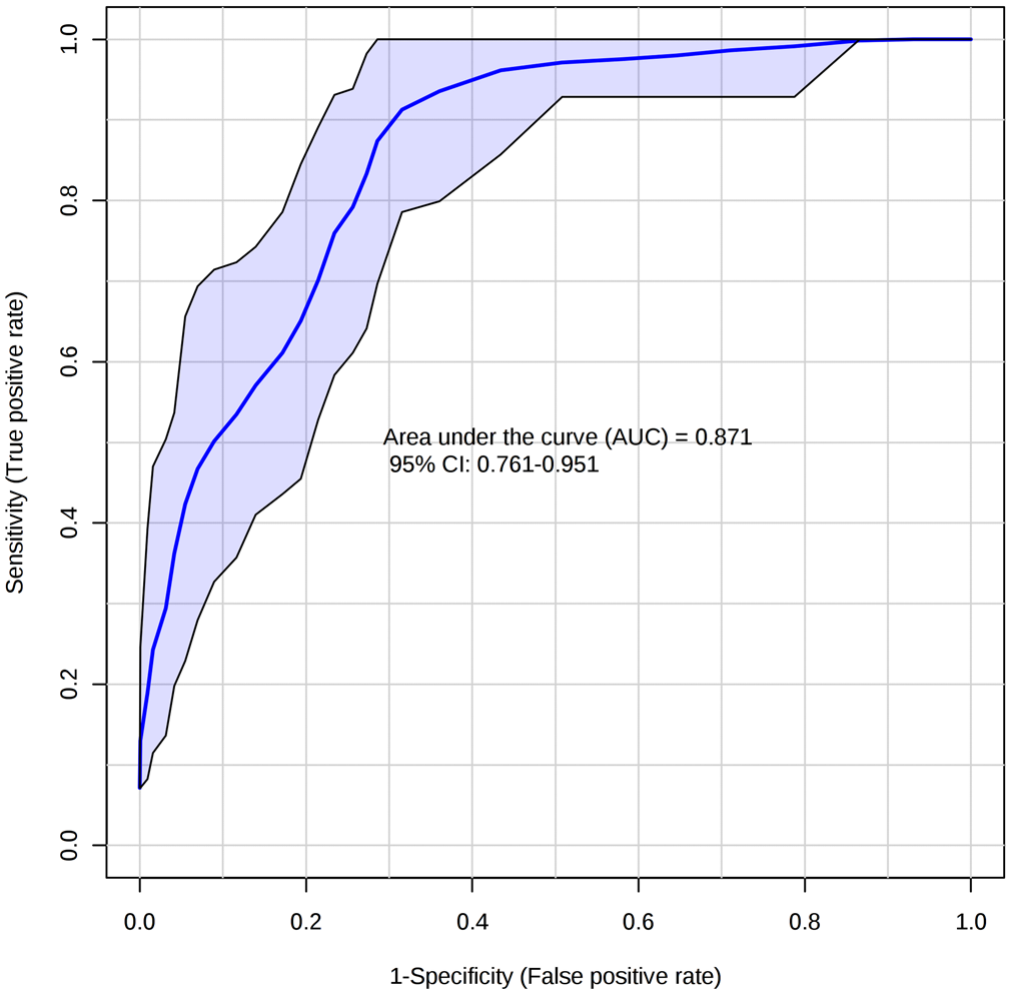
Multi receiver-operating characteristic curve of lactate and 8-isoPGF_2α_ in patients with heart failure. Area under the curve of 0.871 (95% confidence interval: 0.761 to 0.951, *p* < 0.001).

The area under the curve (AUC) of salivary lactate and 8-isoPGF_2α_ were 0.719 (95% confidence interval: 0.607 to 0.831, *p* < 0.001) and 0.661 (95% confidence interval: 0.542 to 0.767, *p* < 0.05), respectively. The combination of both analytes (Fig. 3), resulted in a significant increase in the AUC up to 0.871 (95% confidence interval: 0.761 to 0.951, *p* < 0.001), thus showing an incremental capability to discriminate admission (acute phase) and discharge point.

## Discussion

Isoprostanes represent a novel class of compounds produced *in vivo* from the oxidative peroxidation of arachidonic acid, whereas cortisol is a well know marker of chronic stress related to the hypothalamic-pituitary-adrenal axis activity. In particular, 8-isoPGF_2α_ is considered a representative example of these compounds and a reliable biomarker of free radical mediated lipid peroxidation *in vivo*^48^. Such biomarkers are commonly determined in biological fluids by enzyme-linked immunosorbent assays (ELISA)^44,49^, although commercially available kits show drawbacks related to antibody cross-reactivity which lead to partially inaccurate results^50^. A solid phase extraction (SPE) procedure coupled to well-established LC-MS or GC-MS methods may overcome these issues^51,52^, but the need of larger volumes of sample represents a clear disadvantage, especially in the case of saliva analysis. Micro-extraction techniques such as MEPS overcome this limitation and improve the analytical performance of the MS methods. Our results showed that MEPS is a good alternative to classical clean-up procedures, with a low consumption of organic solvents (<100 µL/measurement), a reusable sorbent (over 100 extractions with the same cartridge) and reduced the cost of analysis. In addition, the MEPS approach permits to automate the extraction procedure, thus enhancing the reproducibility of the analytical method. Compared to other methods^50,53,54^, our procedure showed a satisfactory recovery (95–110%) and an adequate detection limit (10 pg/mL), which allowed a reliable determination of 8-isoPGF_2α_ and cortisol in real samples at the low concentration levels expected in saliva samples.

Validated procedures were used to monitor over time a panel of potential biomarkers such as lactate, uric acid, TNF-α, cortisol, α-amylase and 8-isoPGF_2α_ in saliva samples collected from 44 HF patients during their hospitalization. We used stepwise regression to fit a linear model on thirty-three variables to predict blood NT-proBNP level. In this way, the variables of each saliva sampling were assumed as an independent input vector, thus leading to a 193-by-33 input data set and an output variable. Our model only consisted of those variables with *p*-value < 0.05 whereas variables with *p*-value > 0.1 were removed. We forced the linear model to include only a linear term for each variable and a constant term. The stepwise regression allowed to identify lactate and 8-isoPGF_2α_ as potential predictors of NT-proBNP, which represent one of the most promising HF biomarker^55,56^. The results are in line with the clinical conditions observed in the acute phase and disease severity, most patients being (about 70%) in NYHA class III (n = 18) and IV (n = 14) at hospital admission. The acute phase of HF is characterized by a reduced ability of the heart to pump blood that leads to a decreased perfusion of organs and tissues^57^. In the attempt to restore an adequate perfusion, several neurohormonal mechanisms are activated. Natriuretic peptides are released from the myocardium, increasing the excretion of water and electrolytes and inducing vasodilation^58^. Although beneficial in the early stages of HF, these compensatory mechanisms often lead to a vicious cycle worsening HF^57^. The reduced tissue perfusion increases anaerobic glycolysis, i.e. the conversion of pyruvate to lactate^14^. Plasma lactate levels result from the balance between production (e.g. in skeletal muscle) and clearance, mainly due to liver metabolism (about 70%) and kidney removal^59^. The reduced cardiac output following HF limits the ability of liver and kidney to remove lactate and contributes to increase lactate plasma levels. An additional contribution comes from oxidative stress, which induces subtle changes in intracellular pathways and redox signalling, and causes cellular dysfunction and damage^15^. In addition, the presence of ROS activates a broad variety of pathways with the induction of apoptosis^60^.

The decrease of lactate and 8-isoPGF_2α_ concentrations during hospitalization are likely related to the improved clinical conditions due to treatment. In fact, all patients received diuretics and β-blockers in order to stabilize their clinical conditions and minimize damages to target organs. The improvement of clinical conditions was confirmed by the concomitant decrease of blood NT-proBNP level. Patients suffering from both HF and other comorbidities showed the same decreasing trend of salivary lactate and 8-isoPGF_2α_ and, then, they were indistinguishable from the other patients. As a consequence, it is possible to hypothesize that salivary levels of both lactate and 8-isoPGF_2α_ could be mainly related to cardiac decompensation rather than other conditions. These results were further confirmed when multi ROC curve was built to assess the capability to distinguish admission (acute phase) and discharge point. In fact, a combination of ROC curve analyses of lactate and 8-isoPGF_2α_ showed better sensitivity and specificity, confirming that the concomitant determination of such compounds has a potential value in HF monitoring.

These observations are consistent with the hypothesis that lactate levels and 8-isoPGF_2α_ could be useful in guiding the treatment of acute HF. In addition, assuming that the high lactate and 8-isoPGF_2α_ salivary levels observed in acute conditions build up progressively over time, their monitoring would allow to prevent acute conditions. The avoidance of acute conditions and hospital readmissions would improve the quality of life and possibly prolong survival.

We conclude that this pilot study identified lactate and 8-isoPGF_2α_ as possible non-invasive biomarkers for HF monitoring, and that saliva analysis has potential applications in clinical practice.

## Materials and methods

### Chemicals and materials

Lactate (TraceCERT, 11.10 ± 0.02 mM), 9-chloromethyl-anthracene (purity ≥98%), tetra-n-butylammonium bromide (purity ≥98%), triethanolamine (purity ≥99%), uric acid (purity ≥99%), sodium hydroxide (purity ≥98%, pellets anhydrous), and hydrocortisone (purity ≥98%) were purchased from Sigma Aldrich (Milan, Italy).

8-isoPGF_2α_ and 8-isoPGF_2α_-d4 were purchased at purity ≥99% from Cayman Chemical (Ann Arbor, Michigan, USA).

Phadebas^®^ Alpha-Amylase Test was provided by Magle AB Life Sciences (Sweden).

TNF-α was quantified using the Quantikine^®^ ELISA human TNF-α immunoassay (Cat. No. DTA00D) from R&D Systems (United States). Acetonitrile, methanol, and water at LC-MS grade (purity ≥99.9%) were from Fluka (Milan, Italy). Type I ultrapure water (18.2 MΩ-cm) for ELISAs was obtained using the Elga PURELAB Classic system (France).

All the liquid solutions and saliva samples were stored in sterile polypropylene containers from Eppendorf (Milan, Italy).

Phenex™-RC syringe filters (0.2 μm regenerate cellulose, 4 mm of diameter) were from Phenomenex (California, USA).

Salivette roll-shaped polyester swabs were purchased from Sarstedt (Nümbrecht, Germany).

A Macherey Nagel pehanon narrow range (6.0 < pH < 8.1) pH paper strips (Düren, Germany) with a resolution of 0.3 pH units was used to estimate the salivary pH.

### Preparation of standard solutions and quality control samples

Stock solutions of triethanolamine (265 mM), tetra-n-butylammonium bromide (90 mM), 9-chloromethyl-anthracene (10 mM) were prepared by dissolving the required amounts of the pure compounds in acetonitrile. A stock solution of uric acid (970 μg/mL) was prepared by dissolving a weighed amount of the pure compound in ultrapure water and adding NaOH (10 M). On the same way, a stock solution of cortisol (1000 μg/mL) was prepared by dissolving the appropriate amount of the pure compound in acetonitrile. A stock solution of 8-isoPGF_2α_ (1000 µg/mL) was prepared by dissolving 1 mg of pure compound in 1 mL of a mixture of acetonitrile and methanol (50:50 v/v) and stored at −20 °C in amber vials for 12 months. A stock solution of 8-isoPGF_2α_-d4 in methyl acetate (100 µg/mL) was stored at −20 °C for 12 months.

Working solutions of lactate, uric acid, and cortisol were prepared gravimetrically by an appropriate dilution of the stock solutions with LC-MS water. All stock and working solutions were stored in amber vials at 4 °C and prepared monthly. Working solutions, containing 8-isoPGF_2α_ in the range 0.05–1.25 ng/mL, were prepared through sequential dilutions with LC-MS water from an intermediate stock solution of the analyte, monthly prepared, at 5 µg/mL in a 50:50 v/v acetonitrile:methanol mixture. A working standard mixture of 8-isoPGF_2α_-d4 (20 ng/mL) was prepared through sequential dilutions with LC-MS water from the stock solution. All working solutions were prepared weekly and stored at 4 °C.

Pooled saliva samples were obtained by mixing known aliquots of stimulated saliva samples collected from 20 nominally healthy volunteers. This pooled sample was spiked daily with lactate (i.e. 0.56, 1.10, 2.20 and 5.50 mM), uric acid (i.e. 0.5, 1, 2, 5, 10 and 20 µg/mL), TNF-α (i.e. 4, 8, 16, 31, 63, 125, 250, 500, and 1000 pg/mL), cortisol (i.e. 0.5, 2.5, 5, 10 and 12.5 ng/mL), and 8-isoPGF_2α_ (i.e. 0.05, 0.25, 0.50, 1.00 and 1.25 ng/mL) to obtain standard saliva samples at different concentrations.

### Equipment

A VELP Scientifica ZX4 Advanced Vortex Mixer (Usmate, Italy) and an Eppendorf Centrifuge 5804 R equipped with an A-4-44 swinging bucket rotor (Milan, Italy) were used to homogenize and centrifuge the samples, respectively. A Julabo SW22 thermostatic water bath (Milan, Italy) was used to control the temperature (resolution of 0.1 °C). A Lambda 25 Perkin Elmer UV– Visible spectrophotometer (Milan, Italy), equipped with two quartz cuvettes with an optical path of 1 cm, was used to determine α-amylase.

The amount of the collected saliva sample was calculated according to weight differences before and after sampling by using a Radwag AS220/X balance (Milan, Italy).

Lactate-9-chlormethyl-anthracene adduct and uric acid were analysed by an Agilent 1290 Infinity II LC system coupled to a diode array detector (DAD) and a fluorescence detector (FLD). Chromatographic separation of lactate-9-chlormethyl-anthracene adduct was carried out using a Poroshell 120 EC-C18 reversed-phase column (50 × 2.1 mm, 2.7 μm) connected to an EC-C18 guard column (5 × 2.1 mm, 2.7 μm), both purchased from Agilent Technologies (Santa Clara, USA). Chromatographic separation of uric acid was carried out with a Zorbax SB-Aq reversed-phase column (250 × 4.6 mm, 5 μm) connected to a Zorbax SB-Aq guard column (12.5 × 4.6 mm, 5 μm), both purchased from Agilent Technologies (Santa Clara, USA). The instrument was controlled by OpenLAB software (v. A.01.05) from Agilent Technologies (Santa Clara, USA).

A Thermo Scientific MultiSkan GO microplate reader (Mod. 1.01.12) from Thermo Fisher Scientific (Italy) was used to measure absorbance (in term of optical density, OD) for TNF-α quantification. Cortisol, 8-isoPGF_2α_ and 8-isoPGF_2α_-d4 were analysed by an Agilent 1290 Infinity II LC system coupled to a 6495 Triple Quadrupole mass spectrometer equipped with a Jet Stream electrospray (ESI) ionization source (Agilent Technologies, Santa Clara, USA). Chromatographic separation was achieved using a Polaris 3 C18-A (50 × 4.6 mm, 3 µm) column equipped with a Polaris 3 C18-A MetaGuard column (10 × 4.6 mm, 3 µm), both purchased from Agilent Technologies (Santa Clara, USA). The instrument was controlled by MassHunter Workstation software (B.07.00) from Agilent Technologies (Santa Clara, USA).

8-isoPGF_2α_, 8-isoPGF_2α_-d4 and cortisol were extracted from saliva by an off-line semi-automated MEPS procedure using an eVol® XCHANGE analytical syringe (20–500 µL) coupled to a barrel insert and needle assembly, purchased from SGE Analytical Science (Melbourne, Australia), containing ~4 mg of silica-C18 stationary phase^61,62^. An AL-4000 programmable syringe pump (World Precision Instrument, Sarasota, USA) and a graphical interface, developed in LabView (National Instruments, Austin, USA), was used to perform the entire MEPS procedure.

### Study population

The pilot study was carried out in the framework of HEARTEN project (‘A co-operative mHEALTH environment targeting adherence and management of patients suffering from HF’, Grant agreement: 643694) and was approved (protocol number: 643694) by the Ethics Committee of the Area Vasta Nord-Ovest (CEAVNO-Tuscany Region, Italy). The study was conducted in accordance with the 1964 Declaration of Helsinki and subsequent updates. The study population (36 males, 8 females) consisted of patients hospitalized at the Cardio-Thoracic-Vascular Department of the Azienda Ospedaliera-Universitaria Pisana (AOUP). All subjects gave written informed consent after receiving appropriate information on the protocol. Inclusion criteria for this pilot study were: age ≥18 years and acute HF as the primary cause of hospitalization, which diagnosis was based on the European Society of Cardiology (ESC) guideline criteria^1^. Patients presenting with acute HF from reversible causes (e.g. myocarditis) were not considered, as well as patients who needed intubation. After inclusion, a patient interview was carried out to collect demographics, clinical history, co-morbidities, previous therapies and physical findings. Later on, HF patients were categorized into NYHA functional class based on clinical symptoms identified by the cardiologist. During hospitalization, patients were treated in accordance with the attending physicians’ recommendations of the ESC guidelines^1^.

### Sample collection and analysis

Supplementary Fig. S1 shows a typical five-points sampling timing for the monitor of HF patients during hospital stay. Stimulated saliva samples were collected by asking the patients to roll a Salivette polyester swab in the mouth for 2 min at the time of hospital admission (t_0_), after every 2 days, and up to at the discharge (t_d_). Blood samples were drawn at the admission and at the discharge to perform routine clinical analysis, as suggested by the ESC guidelines^1^.

Circadian cycle effects and interference from fasting were minimized by collecting samples always in the morning (10.00 to 12.00 AM), 2 h after breakfast. The number of the hospitalizations days was not the same for all patients, on average, each stay lasted approximately 5 days.

After sample collection, salivary pH was measured using a narrow range pH paper. The salivary flow rate (millilitres per minute) was calculated from the ratio between the weight difference (g) of the sampling device before and after sampling and the collection time (min), considering the density of sample equal of 1 g/mL^63^. The sample was recovered by centrifuging the swab at 7000 rpm and 4 °C for 5 min. Saliva was then stored in a polypropylene tube at −80 °C until use, with a maximum storage time of 3 month. Prior to analysis, samples were thawed at room temperature (25 ± 2 °C) and then vortex-mixed for ten seconds to homogenize the sample. All the details of the analytical methods are reported in the supplementary information.

### Statistical analysis

The distribution of variables was tested for normality by the Shapiro-Wilk test. Continuous variables with a normal distribution were reported as mean ± standard deviation, whereas variables with skewed distribution were described by median with lower (25^th^ percentile) and upper (75^th^ percentile) quartiles. The potential differences between groups were evaluated using the t-test and Mann-Whitney test. The statistical relationship between demographic and clinical variables were examined by Pearson’s correlation. A two-tailed *p*-value of <0.05 was considered statistically significant.

All data were analysed using GraphPad Prism (v. 8.0) from GraphPad Software Inc. (La Jolla, USA) and MetaboAnalyst 4.0 (https://www.metaboanalyst.ca).

## Supplementary information

Supplementary information.
