# Supplementary material for: Salivary lactate and 8-isoprostaglandin F2α as potential non-invasive biomarkers for monitoring heart failure: a pilot study
Source: Sci Rep. 2020 May 4;10:7441. doi: 10.1038/s41598-020-64112-2 (PMC7198483; doi:10.1038/s41598-020-64112-2)
Supplement: Supplementary file 1 — Supplementary information. [file 41598_2020_64112_MOESM1_ESM.docx]

**Supplementary information**

**Salivary lactate and 8-isoprostaglandin F_2α_ as potential non-invasive biomarkers for monitoring heart failure: a pilot study**

Silvia Ghimenti^1^, Tommaso Lomonaco^1,*^, Francesca G. Bellagambi^1,2^, Denise Biagini^1^, Pietro Salvo^3^, Maria G. Trivella^3^, Maria C. Scali^4^, Valentina Barletta^4^, Mario Marzilli^4^, Fabio Di Francesco^1^, Abdelhamid Errachid^2^ & Roger Fuoco^1^

^1^ Department of Chemistry and Industrial Chemistry, University of Pisa, Via Giuseppe Moruzzi 13, 56124, Pisa, Italy.

^2^ Institut de Sciences Analytiques (ISA) – UMR 5280, Université Claude Bernard Lyon 1, 5 rue de la Doua, 69100, Lyon, France.

^3^ Institute of Clinical Physiology, CNR, Via Giuseppe Moruzzi 3, 56124, Pisa, Italy

^4^ Department of Surgical, Medical and Molecular Pathology and Critical Care Medicine, University of Pisa, Via Paradisa 2, 56124, Pisa, Italy.

^*^ Corresponding Author

Telephone: +39 050-221-9248

Fax: +39 050-221-9260

E-mail address: tommaso.lomonaco@unipi.it

ORCID ID: http://orcid.org/0000-0002-1822-7399

| **Variables** | ***p*-value** |
| --- | --- |
| Lactate | <0.001 |
| 8-isoPGF_2α_ | 0.001 |
| Left ventricular ejection fraction | <0.001 |
| Oxygen saturation | 0.001 |
| Estimated glomerular filtration rate | <0.001 |

**Table S1.** Significant variables to predict the blood NT-proBNP level after applying stepwise regression to the original data set.


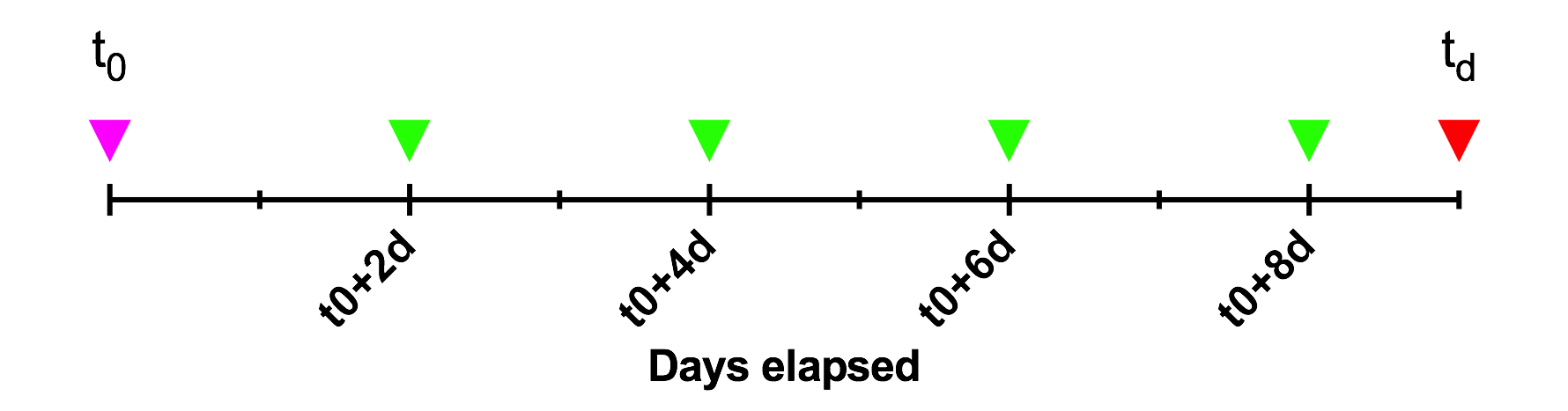


**Figure S1.** A typical sampling schedule. Saliva was collected at hospital admission (purple triangle, t_0_) and up to hospital discharge (red triangle t_d_). Green triangles are referred to the samples collected every 2 days during hospitalization.

**Analytical methods**

**Determination of lactate.** The concentration of lactate in saliva was determined using the analytical procedure reported elsewhere^36^. Briefly, an aliquot of sample (10 μL) was added to an amber reaction vial together with 20 μL of triethanolamine (265 mM), 90 μL of tetra-n-butylammonium bromide (90 mM) and 370 μL of 9-chloromethyl-anthracene (10 mM). The resulting solution (final volume of 490 μL) was incubated at 70 ± 1 °C for 60 min in a thermostatic water-bath in the dark. The solution was cooled for 1 min in ice and diluted (10-fold) with a water-acetonitrile mixture (80:20, v/v). This latter solution was filtered (0.2 μm regenerate cellulose) and then injected (1 μL) into the UHPLC system. Chromatographic separation was performed at 0.5 mL/min using a mobile phase composed by LC-MS water (A) and acetonitrile (B). The composition of mobile phase changed over time as follows: isocratic conditions for 1 min (25% of B), followed by an increase of B at 37% in 13 min. Excitation and emission wavelengths were set at 365 and 410 nm, respectively. The high performance well-plate auto-sampler and thermostat column compartment was set at a temperature of 4 and 25 °C, respectively.

**Determination of uric acid.** The concentration of uric acid in saliva was determined using the analytical procedure reported elsewhere^36^. Briefly, an aliquot of sample (20 μL) was diluted to 1 mL with a water solution containing 0.1% (v/v) formic acid and 3% acetonitrile. The resulting solution was mixed for 30 s and then injected (10 μL) into the UHPLC system. Chromatographic separation was carried out in isocratic mode at 25 °C and 1 mL/min with a mobile phase consisting of 3% acetonitrile and 97% of 0.1% (v/v) aqueous formic acid. Ultraviolet detection was performed at a wavelength of 290 nm. The high performance well-plate auto-sampler and thermostat column compartment was set at a temperature of 4 and 25 °C, respectively.

**Determination of TNF-α.** TNF-α was determined using the ELISA kit sold for its quantification in cell supernates and in human serum and plasma, following the assay procedure provided from the supplier with the exception of the standard curve, which was prepared by spiking PSP with appropriate amounts of analyte in a concentration range of 4−1000 pg/mL. Briefly, after the addition of 50 µL of a buffered protein base with preservatives (Assay Diluent RD1F), 50 µL of sample, standard, or control were incubated for 2 hours at room temperature (22 ± 2 °C) into the well of a polystyrene microplate that was coated with a monoclonal antibody specific for human TNF-α. The microplate was laid on a horizontal orbital microplate shaker set at 80 rpm. After that, each well was aspirated and washed with 400 µL of a buffered surfactant with preservative (Wash Buffer) for a total of four washes, completely removing the liquid each time. After the lash washing, 200 µL of polyclonal antibody specific for human TNF-α conjugate to horseradish peroxidase with preservatives (Human TNF-α Conjugate) were added into each well and incubate for 2 hours at room temperature on the horizontal shaker (80 rpm). At the end of the incubation time, the well washing was repeated as previously described and then 200 µL of Substrate Solution were added to each well. The latter reagent was prepared by mixing, within 15 min of use, equal volumes (e.g. 12 mL) of stabilised hydrogen peroxide and stabilised tetramethylbenzidine. The wells were then incubated for 30 min on a benchtop and covered by an aluminium sheet to be protected from the light. The last step was the subsequent addition of 50 µL of 2N sulphuric acid (Stop Solution). The OD was determined within 30 min using the microplate reader set to 450 nm. To correct possible optical imperfections in the plate, reading at 570 nm was subtracted from the reading at 450 nm.

**Determination of** 𝛂**-amylase.** The activity of the α-amylase was measured as reported elsewhere^31^. Briefly, an aliquot of sample (20 μL) was diluted at 4 mL with fresh LC-MS water and vortex-mixed for 30 s and then incubated at 37.0 ± 0.1 °C. After 5 min, a Phadebas^®^ Alpha-Amylase tablet was insert into the reaction vial and then incubated at 37.0 ± 0.1 °C for another 15 min. The enzymatic reaction was blocked by adding 1 mL of NaOH (0.5 M). The final solution was filter with a syringe filter (0.2 μm cellulose acetate filtering membrane). The activity of α-amylase was determined by measuring the absorbance of the reaction product at 620 nm.

**Determination of 8-isoprostaglandin F_2α_ and cortisol.** The concentration of 8-isoprostaglandin F_2α_ and cortisol was determined using a modified version of a method reported elsewhere^62^. Briefly, an aliquot of saliva (500 μL) was added with 0.01 mL of 8-isoPGF_2α_-d4 (20 ng/mL) and then diluted to 3 mL with water. The resulting mixture was mixed using a vortex mixer for 30 s, filtered with a syringe filter (0.2 µm) and then subjected to MEPS procedure prior to UHPLC-MS/MS analysis. The cartridge was activated by drawing and discharging three times an aliquot (100 µL) of methanol and then conditioned by drawing and discharging three times an aliquot (100 µL) of water. After that, an aliquot (500 µL) of the diluted sample was loaded up and discharged six times in order to retain the target analytes. The cartridge was then washed one time with an aliquot (100 µL) of a water:methanol mixture (95:5 v/v) to remove potential interferences from the samples and to minimize the matrix effect. Finally, the analytes were eluted from the cartridge with one aliquot (50 µL) of pure methanol and then 20 μL of the extracted sample were injected into the UHPLC-MS/MS instrument. Sampling and elution were performed at the lowest flow rate possible (0.3 mL/min), whereas all the other steps at 0.6 mL/min.

The chromatographic separation was achieved using a combination of A (H_2_O + 0.1 % formic acid) and B (50:50 v/v methanol:acetonitrile) at 25 °C and at a flow rate of 0.7 mL/min. The composition of mobile phase was changed over time as follows: isocratic conditions at 10% (B) for 2 min, 45% (B) in 0.1 min, isocratic for 3 min, from 45 to 60% (B) in 3.5 min, isocratic for 0.5 min, 80% (B) at 9 min, isocratic up to 14 min, re-equilibration to initial conditions up to 18 min The Agilent 1290 high performance well-plate auto-sampler was set at a temperature of 4 °C, whereas the Agilent 6495 Triple Quadrupole operated in multiple reaction monitoring (MRM) with unit mass resolution. Nitrogen was used at purity of 99.5% and 99.999% for the ESI source and for the collision gas, respectively. The ESI operation conditions were optimized through the specific tool (MassHunter Optimizer) from Agilent and resulted: drying gas temperature 240 °C, drying gas flow 18 L/min, nebulizer gas pressure 35 and 30 psi respectively for cortisol and 8-isoPGF_2α_, sheath gas temperature 360 °C, sheath gas flow 12 L/min, capillary voltage 3000 and 5000 V respectively for cortisol and 8-isoPGF_2α_ and nozzle 0 and 1500 V respectively for cortisol and 8-isoPGF_2α_. The fragmentor voltage was fixed at 380 V, high and low pressure funnel voltages were set at 160 V for all mass transitions, respectively. Quantifier and qualifier transitions (precursor->product ion) for cortisol were 363->121 (collision energy of 28 eV) and 363->267 (collision energy of 20 eV), respectively. In the case of 8-isoPGF_2α_ were 353->193 (collision energy of 28 eV) and 353->309 (collision energy of 20 eV), respectively and for 8-isoPGF_2α_-d_4_ quantifier and qualifier transitions resulted 357->197 (collision energy of 28 eV) and 357->313 (collision energy of 20 eV), respectively.
